# Analysis of plant gums and saccharide materials in paint samples: comparison of GC-MS analytical procedures and databases

**DOI:** 10.1186/1752-153X-6-115

**Published:** 2012-10-10

**Authors:** Anna Lluveras-Tenorio, Joy Mazurek, Annalaura Restivo, Maria Perla Colombini, Ilaria Bonaduce

**Affiliations:** 1 Dipartimento di Chimica e Chimica Industriale, University of Pisa, Via Risorgimento 35, 56126, Pisa, Italy; 2 Getty Conservation Institute, 1200 Getty Center Dr., Suite 700, Los Angeles, CA 90049, USA

**Keywords:** GC-MS of saccharide materials, Saccharide materials database, Paint binders, Cultural heritage

## Abstract

**Background:**

Saccharide materials have been used for centuries as binding media, to paint, write and illuminate manuscripts and to apply metallic leaf decorations. Although the technical literature often reports on the use of plant gums as binders, actually several other saccharide materials can be encountered in paint samples, not only as major binders, but also as additives. In the literature, there are a variety of analytical procedures that utilize GC-MS to characterize saccharide materials in paint samples, however the chromatographic profiles are often extremely different and it is impossible to compare them and reliably identify the paint binder.

**Results:**

This paper presents a comparison between two different analytical procedures based on GC-MS for the analysis of saccharide materials in works-of-art. The research presented here evaluates the influence of the analytical procedure used, and how it impacts the sugar profiles obtained from the analysis of paint samples that contain saccharide materials. The procedures have been developed, optimised and systematically used to characterise plant gums at the Getty Conservation Institute in Los Angeles, USA (GCI) and the Department of Chemistry and Industrial Chemistry of the University of Pisa, Italy (DCCI). The main steps of the analytical procedures and their optimisation are discussed.

**Conclusions:**

The results presented highlight that the two methods give comparable sugar profiles, whether the samples analysed are simple raw materials, pigmented and unpigmented paint replicas, or paint samples collected from hundreds of centuries old polychrome art objects. A common database of sugar profiles of reference materials commonly found in paint samples was thus compiled. The database presents data also from those materials that only contain a minor saccharide fraction. This database highlights how many sources of saccharides can be found in a paint sample, representing an important step forward in the problem of identifying polysaccharide binders in paint samples.

## Introduction

Plant gums have been used in a variety of applications such as in food emulsifiers, stabilisers, and thickeners, pharmaceuticals, cosmetics, textiles, and in art. Plant gums have been used for centuries as binding media, to paint, write and illuminate manuscripts and to apply metallic leaf decorations [1]. Gums and other kind of saccharide materials, such as honey, fig milk or starch, are known to have been used as binding media, sizing agents or mummification materials since antiquity. Actually, carbohydrates are contained in a variety of materials used as support, binders and varnishes in painted objects [2]. Wood and paper are common paint supports, and carbohydrates, both free and bound, can be encountered as minor fractions in a variety of paint materials, such as proteinaceous binders, as well as plant and animal terpenoid resins.

GC-MS analytical techniques are routinely used to determine the sugar composition of polysaccharide materials, and are among the best suited for the identification of natural organic materials used in the field of Cultural Heritage. GC-MS is a sensitive technique and highly suitable for the analysis of natural organic substances when the resolution and determination of the molecular profile is essential in order to identify the materials present and the ageing pathways [3]. In general the GC-MS analysis of polysaccharide materials requires a chemolysis step, followed by derivatisation [4,5]. Moreover the analysis of saccharide materials in paint samples needs an additional step of purification, in order to remove pigments and fillers. There are many different chemolysis procedures used to study plant gums [6], based on: the methanolysis [7,8], on the hydrolysis [9-13], and hydrolysis assisted by microwaves of the polysaccharide [14-16]. Rates of degradation differ for each monosaccharide during hydrolysis. The sugars are released in the order of ease of bond fission: furanosidic > pyranosidic,6-deoxyhexosidic > hexosidic > and neutral hexosidic > uronosidic [17]. Hydrolysis is complicated if there are proteins or polyphenols in the gum sample, and therefore interaction with the reducing sugars may take place. If the polysaccharide has a limited solubility, this also increases the difficulties. Derivatisation is fundamental in the GC-MS analysis of saccharides, due to the high number of polar moieties present in each molecule [4,18,19]. Thermally assisted hydrolysis and methylation [20] and on-line pyrolysis/silylation [21,22] can also be used, but the interpretation of the data in samples from Cultural Heritage is more complex and needs further investigation. Most sugars in saccharide materials used in the field of Cultural Heritage occur as three types: aldoses (e.g. glucose), ketoses (e.g. fructose), or uronic acids (e.g. glucuronic acid). It is very difficult to analyze all three types of sugars in one quantitative analysis without creating multiple derivatives of each sugar [23]. To avoid the formation of multiple derivatives, which occur because sugars have different isomeric forms in solution [24], various derivatisation procedures have been proposed, such as the reduction of carbonyl moieties followed by acetylation [25,26] the conversion of monosaccharides into acyclic oximes, followed by silylation [11] or acetylation [9,10], or the formation of diethyl mercaptal derivatives followed by silylation [13,27]. Each of these methodologies has its own advantages and drawbacks [1], but none of them is able to derivatise aldoses, ketoses and uronic acids at the same time without inducing any degradation, or producing chromatograms too complex to be unequivocally interpreted.

Because of these inherent difficulties, those analyzing carbohydrates in the field of cultural heritage are not able to fully compare data obtained from a variety of different analytical methodologies, because each of them produce extremely different chromatographic profiles and multiple databases [13,28,29]. Moreover, in most cases the results of the quantitative analyses are not given, so that evaluating the sugar profiles of the paint samples are even more complex.

In this paper we present a comparison between two different analytical procedures that have been independently developed, optimised and systematically used to characterise plant gums in samples collected from works of art [22,27,29-34]. The research was carried out independently at two laboratories: the Getty Conservation Institute in Los Angeles, USA (GCI) and the Department of Chemistry and Industrial Chemistry of the University of Pisa, Italy (DCCI). The GCI procedure is based on the methoxylamine acetate derivatisation of neutral sugars (aldoses and ketoses) obtained from saccharide materials after hydrolysis. The DCCI procedure is based on the analysis of the mercaptal derivatives of the parent aldoses and uronic acids obtained after hydrolysis assisted by microwaves. Both procedures are used to quantitatively determine the sugar profile obtained by each polysaccharide gum after chemolysis. The data obtained with the two procedures are compared in order to understand if the chromatographic profiles are influenced by the analytical protocols and are affected differently by the sample composition. Given the proven homogeneity of the data obtained with the two techniques, a common database of sugar profiles of organic materials which can be encountered in paint samples, has been developed and is presented here.

## Methods

### Reagents, raw materials and reference solutions

Monosaccharides and uronic acids d-(+)-galactose, l-(−)-fucose, l-(+)-arabinose, l-(−)-ramnose, l-(−)-mannose, D-(+)-xylose, D-(+)-glucose, D-glucuronic acid, D-galacturonic acid monohydrate, d-allose, D-glucuronic acid, 2-deoxy-d-ribose, D-psicose, D-tagatose, myo-inositol and mannitol, used as an internal standard, purity 99%, were obtained from Sigma–Aldrich (Milan, Italy).

Trifluoroacetic acid 99% purity, and anhydrous pyridine were from Fluka (Milan, Italy), ethanthiol (ETSH) 99.5%, sodium azide (NaN_3_) 99.5% and *N,O*-bis(trimethylsilyl) trifluoroacetamide (BSTFA) with and without 1% trimethylchlorosilane (TMCS), were from Sigma–Aldrich. Pyridine Sequanal Grade and Trifluoroacetic Acid Sequanal Grade were from Thermo Scientific. Acetic anhydride was from Supelco Inc. Carbohydrates 1, 31 standards and Methoxyamine Hydrochloride were from Sigma Aldrich. Ethyl Alcohol, Absolute, 200 Proof was from Spectrum Quality Products, Inc. Water, high purity, Chloroform, Burdick & Jackson, for GC&GC-MS analysis, 4 L were from VWR Scientific.

Standard solutions of monosaccharides with concentrations of about 100 ppm were prepared in bidistilled water and 1% sodium azide was added to prevent microbial growth. The solutions were stored at 4°C.

Amberlite MB 6113 mixed bed ion exchange resub (with color indicator) was purchased from Fluka Analytical.

### Samples

The raw materials and reference solutions used in this study are detailed in Table 1. The paint samples analysed were collected from:

– Huaca de La Luna, Peru. Polychromy on wood, 10^th^ century. Sample P-c (white, calcite, 0.9 mg).

– Nefertari Tomb, Luxor, Egypt. Mural painting, 13^th^ century BC. Samples: Nef-y (yellow, 0.7 mg); Nef-r (red, 1.0 mg).

**Table 1:** Description of the raw materials analyzed in the study

**Common name**	**Family and species**	**General origins of plant family**	**Origin**
Acacia sp.	*obtained from the sap of Acacia giraffe trees*	Africa (India has several spp.)	L.A. County Arboretum, USA
Angra (acacia)	*obtained from the sap of Acacia karoo trees*	South Africa	University of Oxford, UK
Tahla (acacia)	*obtained from the sap of Acacia seyal trees*	Senegal to Sudan, Africa	University of Oxford, UK
Gum Arabic	*obtained from the sap of trees Acacia senegal*	Tropical Africa	Sigma
Mesquite	*obtained from the plant Prosopis sp.*	North and South America	Mexico
Almond	*exuded by Prunus amygdalus trees*	Eurasia, North Africa	Cal State Polytechnic, USA
Apricot	*exuded by the trees Prunus armeniaca*	Armenia, India, Greece	Cal State Polytechnic, USA
Cherry	*exuded by the trees Prunus serrulata*	Northern Hemisphere	Cal State Polytechnic, USA
Cherry	*exuded by the trees Prunus Cerasus*	Northern Hemisphere	Opificio delle Pietre Dure, Florence
Peach	*exuded by the trees Prunus persica*	China/Persia	Mogao China, Peach Tree Orchard
Plum	*exuded by the trees Prunus*	Eurasia	Czechoslovakia, Prague
			Opificio delle Pietre Dure, Florence
Tragacanth	*obtained from the sap of the plants Astragalus*	Eurasia and Africa	Sigma
			Kremer
			Local market Egypt
			Local market Persia
			Local market Anatolia
Ghatti	*exuded by the trees Anogeissus latifolia*	India	Sigma
Karaya	*exuded by the plant Sterculia* sp.	India	Sigma
Angico	*exuded by the trees Piptadenia* sp.	Brazil	University of Parana, Brazil.
Cashew	*exuded by the plants Anacardium* sp.	India, South America, Southeast Asia, Africa	EMBRAPA, Brazil
Carageean	extracted from Seaweed	China	Serva Feinbiochemica, Germany
Locust bean	*exuded by the trees Ceratonia sp.*	Mediterranean	Sigma
Guar	*is the endosperm of guar beans, legumen of the plants Cyamopsis tetragonolobus*	India	Sigma
Orchid	*obtained from the fruit of the plants Orchis* sp.	World Wide	Australia
Frankincense	*exuded by the trees Boswellia*	Arabic pensinsula and Africa	Oman
			Somalia
Myrrh resin	*sap of the plants Commiphora*	Arabic pensinsula and Africa	Local market
			Zecchi (Italy)
Mastic	*sap of the trees of the genus: Pistacia; species: Pistacia lentiscus*	Mediterranean region	Zecchi (Italy)
Elephant apple	*Dillenia indica*	India	India, local market
Escobilla	*Sida rhombifolia*	North America	Mexico
Rice Powder	*seed of the plants Oryza sativa*	World Wide	Aiko’s Art Materials, Japan
Mangosteen fruit	*obtained from the fruit of Garcinia mangostana plants*	Southeast Asia	Jahan, India
Nopal Cactus	*obtained from the Opuntia-fiucs indica*	South America	California, USA
Honey	*produced by Apis honey bees*	World Wide	Local market, USA
			Acacia, Italy
			Millefiori, Italy
			Chesnut, Italy
Beeswax	*produced by Apis honey bees*	World Wide	local market, Italy
Propolis	*produced by Apis honey bees*	World Wide	local market, Italy
Cochineal dye	*produced from the scale insects Cochineal (Dactylopius coccus)*	primarily tropical and subtropical South America and Mexico	Mexico, Local market
Henna dye	*produced from the leaves of the plants Lawsonia inermis*	tropical and subtropical regions of Africa, southern Asia, and northern Australasia in semi-arid zones	Kremer
Indigo dye	*extracted from the Indigofera plants*	originally from Pakistan, Indigofera plants can be found in tropical and subtropical regions of the world	Mexico, local market
Redwood dye			Kremer
paper/ wood (average)		World Wide	Unknown
wood	*Beech*	hardwood	Fagus sylvatica
	*Oak*	hardwood	Quercus roburs
	*Pine*	softwood	Pinus sylvestris
	*Fir*	softwood	Abies alba
White fluffy fungus	*obtained from Acremonium* spp.		Mogao Cave, China
Luohanguo	*water extract fruit of the of Siraiti trees; species: Siraitia grosvenorii*	China	local market, China
fig latex	*obtained from Common Fig trees (Ficus carica), when the fruit is detached from the branch*	Mediterranean region, Iran, Pakistan and northern India, and also in other areas of the world with a similar climate	from fig tree, Italy
Hen's Egg		World Wide	local market, Italy
Animal glue	*obtained from the cartilageneous parts of rabbits*	World Wide	Zecchi (Italy)
Cow's Milk		World Wide	local market, Italy

### Analytical procedures

Analytical procedure used at GCI based on the conversion of aldoses and ketoses into acyclic methoximes, followed by acetylation

1. Samples are weighed on the ultramicrobalance and placed in a conical reaction vial. A solution of allose as internal standard is added to give a final concentration of 20 ppm in the injection volume.

2. 100 μl of 1.2 M trifluoroacetic acid is added, oxygen is evacuated under a stream of nitrogen for 30 s. Hydrolysis is performed in the closed vials at 125°C for 1 h. After hydrolysis, the vials are removed from the heat and left to stand until cool.

3. To remove the insoluble inorganic matter, vials are centrifuged, and the supernatant liquid is transferred to a 2 ml autosampler vial. The contents are evaporated using a nitrogen stream while warming the vial to 50°C. Vials are rinsed with 40 μl water, and evaporated under a stream of nitrogen. 40 μl of ethanol is added and evaporated to dryness under a stream of nitrogen.

4. 200 μl of a solution of O-methoxyamine hydrochloride in pyridine and methanol (300 mg/2 ml/1 ml) are added to the vial, and the cap is replaced. The vial is heated at 70°C for 20 min, and is then removed from the heat and left to stand until cool. The solution is slowly evaporated for 10 min to syrup.

5. 400 μl of acetic anhydride in pyridine (3 ml/1 ml) is added and the cap is replaced. The vial is kept at 70°C for 20 min; it is then removed from the heat, left to stand until cool, and the solution is evaporated using a nitrogen stream to syrup or dryness. The contents are reconstituted in 400 μl of chloroform.

6. To remove salts and pyridine the chloroform solution is rinsed twice with 500 μl 0.1 M hydrochloric acid and 500 μl of deionised water. The chloroform solution is removed between rinsing and reduced under nitrogen stream to 50 μl of chloroform. 1 μl of the chloroform solution containing the acetylated methoximes of the parent sugars is injected into the GC-MS.

Analytical procedure used at DCCI based on the conversion of aldoses and uronic acids into diethyl mercaptal derivatives followed by silylation

1. A weighed amount of the sample is admixed with 200–400 μL of 2.5 M NH_3_ to the sample in a conic glass vial, and is placed in an ultrasonic bath at 60°C for 120 min twice, to remove insoluble organic and inorganic matter. The ammonia solution is transferred into a PTFE closed vial and is dried under a nitrogen stream.

2. 0.5 ml of trifluoroacetic acid 2 M is added, and subjected to microwave-assisted acid hydrolysis (power 500 W, temperature 120°C, duration 20 min).

3. After hydrolysis, the sample is filtered with a PTFE membrane filter and then dried in the rotatory evaporator.

4. Once reconstituted in 100 μl of bidistilled water, the freed sugars are purified on a Amberlite MB 6113 mixed bed ion exchange resin, packed on a 0.5 cm diameter glass column. Sugars are eluted with 1 ml of bidistilled water. Soluble inorganic salts are thus retained in the stationary phase.

5. An aliquot of the solution containing monosaccharides and uronic acids is added with the derivatization internal standard solution, evaporated to dryness in the rotary evaporator and subjected to mercaptalation. By admixing 25 μl of ethanethiol/trifluoroacetic acid (2/1, v/v), and keeping the resulting solution at room temperature for 10 min and shaking it sporadically, the corresponding diethyldithioacetals and diethyl dithioacetal lactones are formed. The mercaptalation mixture is then subjected to silylation prior to GC/MS analysis. The silylation is performed in two steps. In the first step, 100 μl of BSTFA is added to the mercaptalation mixture and the mixture is kept for 15 min at 60°C. The solution is then dried under a nitrogen flow, and subsequently added with 50 μl of BSTFA (1% TMCS) as a derivatising agent and 100 μl pyridine as a solvent, and kept at 60°C for 45 min.

6. The reaction mixture is then dried under a nitrogen flow and reconstituted in 50 μl of hexane: 2 μl of this solution, containing diethyl-dithioacetal trimethylsilyl derivatives of the parent sugars, is then injected into the gas chromatograph.

### Blank evaluation

Environmental contamination must be taken into account especially when samples are collected from the field of cultural heritage. In particular, xylose and glucose are widespread and can show very high contamination levels. To avoid misinterpreting the chromatograms obtained, blank evaluations are thus fundamental to assess whether a sugar is really present in the sample, or whether it belongs to the environmental or laboratory blanks. An environmental blank is a taken from the object, or nearby, where it is expected not to contain gum, and provides an indication of the levels of carbohydrates in the environment; this is especially important when testing samples from mural paintings or outdoor paintings. At DCCI, laboratory blanks are periodically run to determine the detection limit (LOD) and quantitation limit (LOQ) of the analytical procedures for each sugar and to assess whether a sugar is present in a sample or is due to environmental contamination. At GCI a different approach is used: the relative sugar content of a sample, determined as the sum of the absolute amount of detected sugars in a sample, must be above 0.1% of the sample weight, and must be 5X times as concentrated when compared to the environmental blank and have a significantly different profile in order to assess whether or not a sample contains a saccharide binder.

## Results and discussions

### Analytical procedures - a comparison

**Figure 1:**
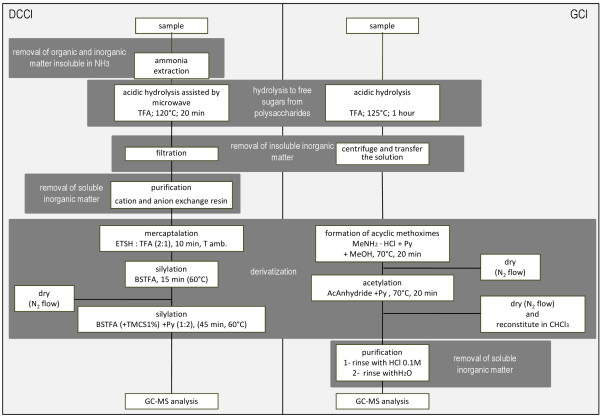
A comparative scheme of the two analytical procedures.

The GC-MS analysis of polysaccharide materials requires a hydrolysis step, followed by derivatisation. A comparison of each analytical step in the two procedures is presented in Figure 1. The two procedures here presented are the evolution of analytical protocols previously reported in the literature [27,35]

As it can be seen in Figure 1, there are three main steps that are required in order to analyse sugars by GC-MS: hydrolysis, derivatisation and purification from inorganic materials. Below, the main steps are discussed and the optimised steps in respect to the published literature are highlighted.

#### Hydrolysis

The hydrolysing agent used in both procedures is trifluoracetic acid (TFA) because it shows a good compromise between reaction efficacy and degradation of most labile sugars. Trifluoroacetic acid is also relatively volatile, meaning that it can be removed from the reaction mixture under a stream of nitrogen. At DCCI microwave assisted hydrolysis was used, as it was proven to significantly shorten reaction time, giving good reaction yields and a reduced decomposition of labile sugars [14]. At GCI hydrolysis is performed at the same temperature than at DCCI, but using conventional heating.

#### Optimization of hydrolysis condition (GCI)

**Figure 2:**
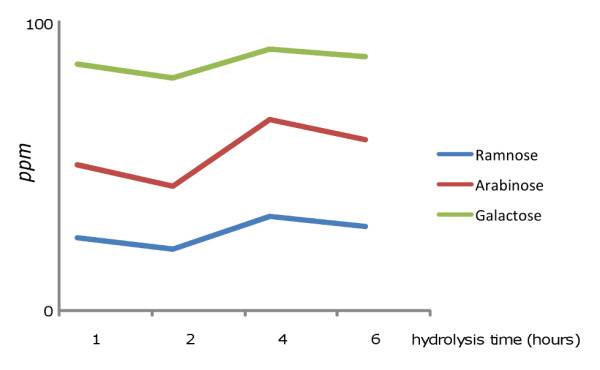
Content of the sugars freed from the hydrolysis of arabic gum, expressed in ppm as a function of the hydrolysis time.

Hydrolysis time was optimized with respect to the literature work [35], in order to find the right compromise between the reaction yield and decomposition of labile sugars which can be encountered in plant gums used in the field of cultural heritage. In particular, 1, 2, 4 and 6 h hydrolysis of arabic gum were tested showing the same behavior for all sugars: the sugars were compared to the yield at 1 h of hydrolysis, and they decreased at 2 h, increased at 4 h, and decreased again at 6 h. This is shown for arabic gum in Figure 2.

The behavior observed at 6 h with respect to 4 h can be explained considering that sugars are degraded after a prolonged heating. The decrease observed after 2 h, followed by an increase at 4 h might be ascribed to a degradation of the sugars freed in the first stage of the hydrolysis, which is subsequently recovered by higher efficacy of the hydrolysis. On the basis of these results, the best hydrolysis duration would be 4 h, but considering that in a sample from works of art free fructose might be present, this is not possible, as fructose is degraded after 2 h exposure to TFA. As a result 1 h hydrolysis was chosen, as it appeared to be the best compromise.

#### Derivatisation

**Figure 3:**
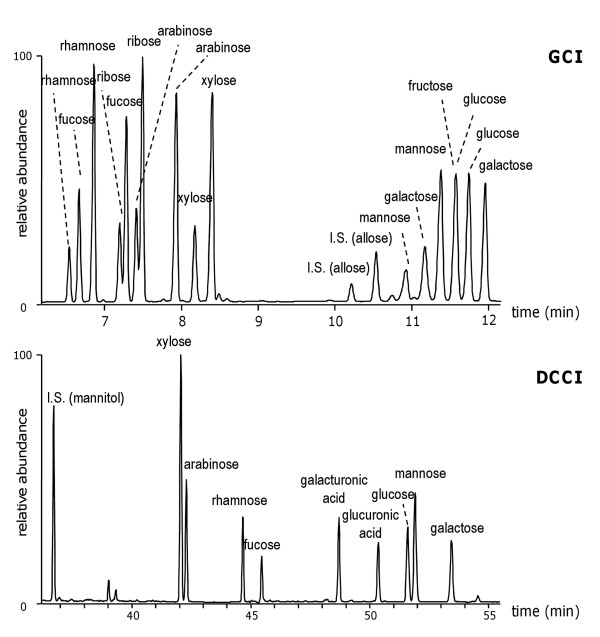
GCI- chromatograms of a standard solution of aldoses and ketoses; DCCI – chromatogram of a standard solution of aldoses and uronic acids.

The GCI procedure is based on the conversion of aldoses and ketoses into acyclic methoximes, followed by acetylation. Two peaks are generated in reproducible ratios in the chromatogram for each sugar: the syn and anti form, and uronic acids are not derivatised. The chromatogram of a standard solution of aldoses and ketoses is reported in Figure 3. The DCCI procedure is based on the conversion of aldoses and uronic acids into diethyl mercaptal derivatives followed by silylation. One chromatographic peak is obtained for each sugar. Ketoses are not quantitatively derivatised producing several peaks in the chromatogram, since ketoses undergo decomposition reactions during mercaptalation. The chromatogram of a stardard solution of aldoses and uronic acids is reported in Figure 3.

Given the high reproducibility of the derivatisation procedures (RSD < 10% for both DCCI and GCI), quantitative analyses are possible.

#### Purification from inorganic materials

The two analytical procedures provide more than one step to remove both soluble and insoluble inorganic materials. Purification from inorganic materials is extremely important in the analysis of paint samples, as pigments and fillers are often major constituents of the paint layers, and may interfere at different levels during the analytical procedure. In order to investigate the possible negative effects of pigments on the GCI derivatization process, a mixture of gum arabic and gum tragacanth were added to 21 different pigments and were analyzed for sugars. Most of the pigments had little to no effect on the sugars: hematite (Fe_2_O_3_), lead white ((PbCO_3_)_2_·Pb(OH)_2_), viridian green (Cr_2_O_3_·2H_2_O), yellow ochre (Fe_2_O_3_·H_2_O), vermillion (HgS), milori blue (C_28_H_14_N_2_O_4_), bone black (C), cadmium red (CdS+CdSe), malachite (Cu_2_CO_3_(OH)_2_), ultramarine (Na_8-10_Al_6_Si_6_O_24_S_2-4_), orpiment (As_2_S_3_), azurite (Cu_3_(CO_3_)_2_(OH)_2_) all showed extremely reproducible chromatograms. A measure of the extent of the reproducibility of the sugar profiles obtained from the pigmented systems with respect to the unpigmented ones can be expressed by calculating the correlation coefficients. For the above mentioned pigments values between 0.99-1.0 were obtained. Several of the pigments showed small effects: zinc white (ZnO), verdigris (Cu(CH_3_COO)_2_·CuO·6H_2_O; 2Cu(CH_3_COO)_2_·CuO·6H_2_O; Cu(CH_3_COO)_2_·H_2_O), red lead (2PbO·PbO_2_), and vine black (C) all gave good matching with the profiles of the reference gums (correlation coefficients were between 0.96-0.98). Some pigments gave poorer matching with the reference gums mainly due to slightly altered quantitative profiles: burnt umber (clay containing oxides of Fe and Mn) (correlation coefficient: 0.93), French ochre (correlation coefficient: 0.93), chalk (CaCO_3_)(correlation coefficient: 0.90), chrome, yellow (PbCrO_4_) (correlation coefficient: 0.90), and calcium sulphate (CaSO_4_) (correlation coefficient: 0.87).

#### Optimization of the purification step (DCCI)

An ammonia extraction step was introduced in the DCCI analytical procedure as a further optimisation of a previously published procedure [29] as it was observed that certain pigments can strongly interfere in the hydrolysis step, leading to chromatographic profiles different from those obtained from the reference materials. Table 2 shows the sugar profiles obtained for a paint replica containing arabic gum and red lead (2PbO·PbO_2_).

**Table 2:** Sugar profile obtained from the paint replicas containing arabic gum and red lead using the procedure with and without ammonia extraction

**Sample**		**Relative percentage content (%)**									**Weight%**
		**xylose**	**arabinose**	**rhamnose**	**fucose**	**galacturonic acid**	**glucuronic acid**	**glucose**	**mannose**	**galactose**	
*paint replica with arabic gum and red lead*	*without ammonia extraction*	9	11	1	0	0	7	1	0	71	0.7*
	*with ammonia extraction*	0	34	15	0	0	12	0	0	39	1.4*
*Reference arabic gum*		0	26	13	0	0	12	0	0	49	65.2*

*UC note that the paint replica gave a much lower recovery of arabic gum, as the sample weigh is dominated by the read led.

The sugar profile obtained without the ammonia extraction is extremely different from that of the reference gum. This suggests that the gum, during hydrolysis, can react with the pigment, maybe with the aid of the microwaves, leading to the formation of unpredictable reaction products, and resulting in chromatographic profile that cannot be related to the literature data. The introduction of the ammonia step completely solves this problem: the same samples, when subjected to ammonia extraction, gave chromatographic profiles that were perfectly in agreement with those of the reference materials.

In addition, for the same reasons, ammonia extraction enabled higher amounts of saccharide material to be recovered from the sample, as indicated by the weight% calculated as the relative weight of the saccharide material (determined as the sum of the quantified sugars) with respect to the sample weight. This is particularly important in painting samples where the amount of organic material can be very low.

#### Comparison of the data obtained with the two analytical procedures

In order to compare the data obtained with the two analytical procedures, the relative sugar percentage composition will be used when analysing paint samples, as the absolute amount of organic content cannot be determined by GC-MS, because the amount of material that has undergone degradation, oxidation and cross-linking reactions cannot be determined. Conversely, saccharide materials are identified on the basis of the sugar profiles [3]. Additional file 1: Table S1 shows the relative sugar percentage content obtained with the two analytical procedures on samples of unpigmented and pigmented arabic, cherry and tragacanth gums. Data presented are the average of triplicate analyses. The average composition and corresponding confidence interval, at a confidence level of 99%, of the tree gums according to both of the procedures is reported in Table 3.

**Table 3:** Average relative sugar percentage content of arabic, cherry and tragacanth gums obtained with the DCCI and GCI procedures, and corresponding confidence interval at a confidence level of 99%

**Gum**	**Relative percentage content (%)**										**Analytical procedure**
	**xylose**	**arabinose**	**rhamnose**	**fucose**	**galacturonic acid**	**glucuronic acid**	**fructose**	**glucose**	**mannose**	**galactose**	
arabic	0	28 ± 4	14 ± 2	0	0	12 ± 3	-	0	0	45 ± 5	DCCI
	0	37 ± 3	18 ± 3	0	-	-	0	0	0	44 ± 5	GCI
cherry	6 ± 3	46 ± 10	1 ± 1	0	0	7 ± 7	-	0	3 ± 2	34 ± 14	DCCI
	11 ± 1	50 ± 6	2 ± 1	0	-	-	0	1 ± 1	2 ± 2	34 ± 6	GCI
tragacanth	22 ± 5	34 ± 6	2 ± 1	8 ± 2	8 ± 9	1 ± 1	-	12 ± 6	0	12 ± 2	DCCI
	24 ± 3	45 ± 5	1 ± 0	11 ± 3	-	-	1 ± 0	8 ± 3	0	9 ± 2	GCI

In order to evaluate whether or not the data obtained with the two procedures are comparable, the mean values of the relative sugar content of each gum were compared by the *t* test., The F-test was used to compare the variances between the two procedures and for the sugars^a^ in each gum, in order to define which formula to use to perform the *t* test. At a confidence level of 99%, the variances obtained for all sugars with the two procedures were not significantly different, with the exception of the relative percentage content of xylose in cherry gum. The unpaired *t* test was, thus, chosen for comparing the mean values of independent samples and equal variance (homoscedastic *t*-test). The confidence levels considered were at 99% and 99.9% level. The calculated values of the t student for each sugar in each gum, and the theoretical values are reported in Table 4.

**Table 4:** Calculated values of t student for each sugar in each gum, and corresponding theoretical values

**Gum**	**t student**	**xylose**	**arabinose**	**rhamnose**	**fucose**	**mannose**	**galactose**
arabic	t theoretical *confidence level: 99%*	2.86093					
	t theoretical *confidence level: 99.9%*	3.8834					
	t calculated	-	−3.3	−2.0	-	-	2.9
cherry	t theoretical *confidence level: 99%*	2.97684					
	t theoretical *confidence level: 99.9%*	4.1405					
	t calculated	−4.3	−0.1	−0.4	-	2.0	1.1
tragacanth	t theoretical confidence level: 99%	3.05454					
	t theoretical *confidence level: 99.9%*	4.3178					
	t calculated	0.5	−3.1	7.3	−2.0	-	4.7

The results indicate that at a confidence level of 99%, 55% of the data belong to the same statistical population, while, if we widen the confidence interval by choosing a confidence level of 99.9%, over 75% of the data appear to belong to the same statistical population. These percentages are surprisingly good if we consider that we have been comparing data available from two independent sets of samples analysed with two procedures, and the experiments were not originally designed to perform this comparison. Moreover, plant gums are characterised by a high intrinsic variabilities. It is a known fact that parts of the same plant or segments of the plant growing in different conditions, seasons, or maturity, may give products that vary in composition and structure [36].

**Figure 4:**
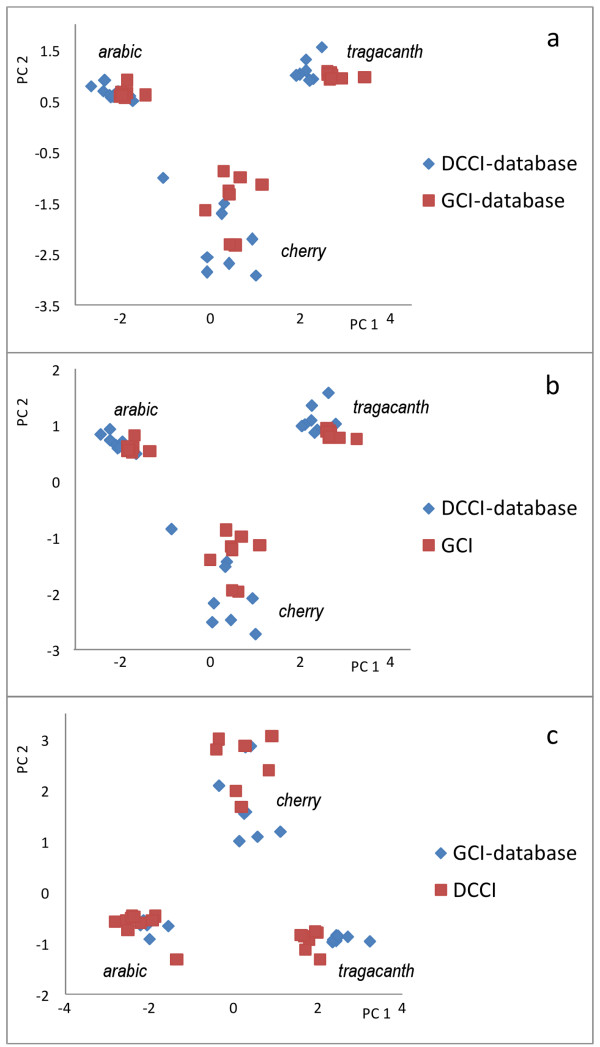
PCA score plots obtained using a) both DCCI and GCI profiles as database; b) DCCI profiles as database and GCI as samples; GCI profiles as database and DCCI as samples.

To further investigate the comparability of the data, Principal Component Analysis was performed on the dataset obtained by combining the DCCI and GCI data, and the score plot is reported in Figure 4 a. The figure clearly shows that the samples are grouped in three clusters, one for each gum. Moreover the data of DCCI and GCI were separately used to build two databases: the data obtained with one procedure was subjected to PCA with the dataset obtained from the other procedure. The resulting score plots are reported in Figure 4b and Figure 4c. The results show that if we compare the sugar profile of a sample obtained with one procedure with the dataset obtained with the other procedure, we obtain a correct assignment of the sample to the corresponding cluster.

All this concur to conclude that the data obtained with the two procedures on reference samples are highly comparable.

Finally, results obtained from the analysis of ancient paint samples were compared. In particular two works of art were analysed: a mural painting from Egypt, 13^th^ century BC, and a polychromy of wood coming from Perù, 10^th^ century AD. Two samples from Egypt were analyzed with the the two procedures, sample Nef-y (containing yellow ochre [37] analzed at the GCI) and Nef-r (containing red ochre [37] analyzed at the DCCI). One sample from a Peruvian wooden painting, (P-c containing calcite) was split into two aliquots and analysed at DCCI and GCI. The samples were collected from paintings with different geographical origins (Peru and Egypt) made with different pigments, were several centuries old, underwent severe ageing conditions, due to temperature and humidity variations, and were subject to different conservation campaigns. The comparison of this data may allow us to understand if the data obtained from the two procedures are still comparable when such complex matrixes are analysed. Given the dimensions and uniqueness of the samples from the paintings, it was not possible to perform replicate analysis on different sample aliquots, and thus it was not possible to perform the *t* test. Data obtained are presented in Table 5.

**Table 5:** Sugar composition of the painting samples collected from the paintings in El Brujo (Peru) and in the Nefertari tomb (Egypt)

**Sample**	**Analytical procedure**	**Sugar relative content**	**Sugar composition (%)**								
			**Xylose**	**arabinose**	**rhamnose**	**fucose**	**galacturonic acid**	**glucuronic acid**	**mannose**	**galactose**	**glucose**
P-c	GCI	0.15%	24	25	4	0	-	-	9	37	Yes
P-c	DCCI	0.12%	17	15	5	1	0	0	14	44	Yes
Nef-y	GCI	2.04%	0	70	1	0	-	-	0	29	No
Nef-r	DCCI	1.54%	0	61	0	0	0	0	0	39	No

Table 5 clearly shows that the sugar profiles obtained for each paint sample using the two analytical procedures are similar. In order to measure this similarity, the correlation coefficients were calculated, resulting 0.98 for the Egyptian samples and 0.90 for the Peruvian one. These results are acceptable if we consider the variability associated with these analytical procedures and the intrinsically inhomogeneous nature of the samples. This is another indication that the GCI and DCCI protocols for the GC-MS analysis of polysaccharide materials produce highly comparable data^b^.

However, the sugar profiles obtained from the paint samples do not match the reference arabic, fruit tree and tragacanth gums, rending problems with PCA results, and underlining the limitations of databases that contain the sugar profiles that we have at our disposal.

### Databases

Given the fact that the GCI and DCCI procedures gave highly comparable data, it has been possible to build a common database of sugar profiles obtained by analysing materials, containing a major or minor saccharide fraction, which can be commonly found in a paint samples. A subset of this database obtained from the analysis of the raw unpigmented reference materials is shown in Table 6.

**Table 6:** Database of the average relative percentage sugar composition of raw unpigmented reference materials

**Common name**	**Family and species**	**General origins of plant family**	**Relative percentage content**										**Total Sugars %**	**Procedure**
			**xylose**	**arabinose**	**rhamnose**	**fucose**	**galacturonic acid**	**glucuronic acid**	**glucose**	**mannose**	**galactose**	**fructose**		
Acacia sp.	*obtained from the sap of Acacia giraffe trees*	Africa (India has several spp.)	0	30	4	0	-	-	0	1	65	0	*22*	GCI
Angra (acacia)	*obtained from the sap of Acacia karoo trees*	South Africa	0	59	2	0	-	-	0	0	39	0	*66*	GCI
Tahla (acacia)	*obtained from the sap of Acacia seyal trees*	Senegal to Sudan, Africa	0	63	0	0	-	-	0	0	36	0	*71*	GCI
Gum Arabic	*obtained from the sap of trees Acacia senegal*	Tropical Africa	0	38	17	0	-	-	0	0	45	0	*65*	GCI
			0	26	13	0	0	12	0	0	49	no	*90*	DCCI
Mesquite	*obtained from the plant Prosopis sp.*	North and South America	0	71	1	0	-	-	0	0	29	0	*77*	GCI
Almond	*exuded by Prunus amygdalus trees*	Eurasia, North Africa	14	49	1	0	-	-	0	1	34	0	*56*	GCI
Apricot	*exuded by the trees Prunus armeniaca*	Armenia, India, Greece	7	44	1	0	-	-	1	4	43	0	*60*	GCI
Cherry	*exuded by the trees Prunus serrulata*	Northern Hemisphere	8	57	1	0	-	-	1	2	31	0	*67*	GCI
Cherry	*exuded by the trees Prunus Cerasus*	Northern Hemisphere	5	35	2	0	0	4	0	2	52	no	*95*	DCCI
Peach	*exuded by the trees Prunus persica*	China/Persia	14	44	2	0	-	-	1	1	38	0	*73*	GCI
			15	44	1	0	0	4	0	2	34	no	*34*	DCCI
Plum	*exuded by the trees Prunus*	Eurasia	11	43	4	0	-	-	0	3	40	0	*75*	GCI
			12	31	3	0	0	13	0	4	37	no	*12*	DCCI
Tragacanth	*obtained from the sap of the plants Astragalus*	Eurasia and Africa	22	43	2	9	-	-	10	0	12	0	*57*	GCI
			19	31	2	6	17	1	12	0	12	no	*50*	DCCI
			19	43	5	7	0	0	12	0	14	no	*24*	GCI
			24	20	2	19	7	0	18	0	10	no	*14*	DCCI
			14	40	6	7	0	0	12	0	21	no	*27*	DCCI
			9	38	5	4	3	0	20	0	21	no	*24*	DCCI
Ghatti	*exuded by the trees Anogeissus latifolia*	India	1	60	2	1	-	-	1	1	34	0	*55*	GCI
			0	47	3	0	0	11	0	2	37	no	*65*	DCCI
Karaya	*exuded by the plant Sterculia* sp.	India	0	1	40	0	-	-	0	0	58	0	*37*	GCI
			0	0	25	0	7	4	0	0	64	no	*41*	DCCI
Angico	*exuded by the trees Piptadenia* sp.	Brazil	0	61	11	0	-	-	0	0	27	0	*75*	GCI
Cashew	*exuded by the plants Anacardium* sp.	India, South America, Southeast Asia, Africa	0	28	1	0	-	-	1	0	70	0	*30*	GCI
Carageean	extracted from Seaweed	China	1	0	0	0	-	-	4	0	95	0	*33*	GCI
Locust bean	*exuded by the trees Ceratonia sp.*	Mediterranean	1	8	0	0	-	-	3	63	21	5	*84*	GCI
			0	0	0	0	0	0	0	62	38	no	*30*	DCCI
Guar	*is the endosperm of guar beans, legumen of the plants Cyamopsis tetragonolobus*	India	0	2	0	0	-	-	2	58	37	1	*91*	GCI
			0	2	0	0	0	0	0	63	35	no	*75*	DCCI
Orchid	*obtained from the fruit of the plants Orchis* sp.	World Wide	0	0	1	0	-	-	25	72	1	0	*62*	GCI
Frankincense	*exuded by the trees Boswellia*	Arabic pensinsula and Africa	3	28	10	2	-	-	11	1	40	5	*1*	GCI
			0	21	9	0	0	6	0	1	62	no	*5*	DCCI
			0	23	1	1	0	3	0	1	72	no	*12*	DCCI
Myrrh resin	*sap of the plants Commiphora*	Arabic pensinsula and Africa	2	30	0	3	-	-	0	8	58	0	*16*	GCI
			3	43	1	1	0	2	0	4	46	no	*7*	DCCI
Mastic	*sap of the trees of the genus: Pistacia; species: Pistacia lentiscus*	Mediterranean region	0	51	2	1	0	2	0	3	40	no	*1*	DCCI
Elephant apple	*Dillenia indica*	India	5	46	0	0	-	-	0	0	49	0	*57*	GCI
Escobilla	*Sida rhombifolia*	North America	13	6	19	0	-	-	36	2	20	4	*14*	GCI
Rice Powder	*seed of the plants Oryza sativa*	World Wide	0	0	0	0	-	-	100	0	0	0	*58*	GCI
Mangosteen fruit	*obtained from the fruit of Garcinia mangostana plants*	Southeast Asia	2	2	1	0	-	-	4	1	2	87	*18*	GCI
Nopal Cactus	*obtained from the Opuntia-fiucs indica*	South America	1	23	4	0	-	-	3	0	69	0		GCI
Honey	*produced by Apis honey bees*	World Wide	0	0	0	0	-	-	31	0	0	69	*56*	GCI
			0	0	0	0	0	0	100	0	0	yes	*20*	DCCI
			0	0	0	0	0	0	100	0	0	yes	*20*	DCCI
			0	0	0	0	0	0	100	0	0	yes	*20*	DCCI
Beeswax	*produced by Apis honey bees*	World Wide	0	0	0	0	0	0	100	0	0	no	*0*	DCCI
Propolis	*produced by Apis honey bees*	World Wide	9	16	13	0	0	1	40	13	8	yes	*2*	DCCI
Cochineal dye	*produced from the scale insects Cochineal (Dactylopius coccus)*	primarily tropical and subtropical South America and Mexico	0	0	0	0	-	-	69	14	1	11	*4*	GCI
Henna dye	*produced from the leaves of the plants Lawsonia inermis*	tropical and subtropical regions of Africa, southern Asia, and northern Australasia in semi-arid zones	7	9	3	0	-	-	61	1	8	10	*8*	GCI
Indigo dye	*extracted from the Indigofera plants*	originally from Pakistan, Indigofera plants can be found in tropical and subtropical regions of the world	1	6	24	1	-	-	33	2	14	4	*2*	GCI
Redwood dye			76	3	2	0	-	-	13	3	4	0	*7*	GCI
paper/ wood (average)		World Wide	70	2	1	0	-	-	20	3	1	1	*8*	GCI
wood	*Beech*	hardwood	75	2	1	0	0	0	16	1	5	no	*7*	DCCI
	*Oak*	hardwood	72	2	0	0	0	0	21	3	2	no	*13*	DCCI
	*Pine*	softwood	24	7	0	0	1	0	27	32	9	no	*7*	DCCI
	*Fir*	softwood	22	4	0	0	0	0	33	36	5	no	*7*	DCCI
White fluffy fungus	*obtained from Acremonium* spp.		1	1	0	0	-	-	22	16	59	0	*15*	GCI
Luohanguo	*water extract fruit of the of Siraiti trees; species: Siraitia grosvenorii*	China	0	0	0	0	0	0	100	0	0	no	*nd*	DCCI
fig latex	*obtained from Common Fig trees (Ficus carica), when the fruit is detached from the branch*	Mediterranean region, Iran, Pakistan and northern India, and also in other areas of the world with a similar climate	3	7	37	4	0	0	23	17	9	no	*nd*	DCCI
Hen's Egg		World Wide	0	0	0	0	0	0	18	70	12	no	*<1*	DCCI
Animal glue	*obtained from the cartilageneous parts of rabbits*	World Wide	2	0	0	0	0	0	35	2	61	no	*<1*	DCCI
Cow's Milk		World Wide	0	0	0	0	0	0	39	3	59	no	*12*	DCCI

The table reports the sugar profiles of different plant gums of different geographical origins, such as a variety of arabic, fruit tree and tragacanth gums, as well as other plant gums which are not known to be used as paint materials, but are never the less produced by plants widespread in areas where arabic, fruit tree and tragacanth are not present. Moreover the table presents the sugar content of materials that were commonly added, by artists or manufacturers, to plant gums in order to modify their physical properties, such as honey, sugar or starch [28]. The sugar profile of “non-saccharide materials” that may be present in works of art is also reported. Organic colorants, proteinaceous binders, and gum resins all contain sugars that contribute to the saccharide content of a sample. Therefore, the detection of sugars in a painting sample can be ascribable not only to a plant gum, but also to different other binders, and as a result, a mixture of organic materials may lead to chromatogram profiles that are difficult to interpret when the overall composition of the organic materials is unknown. In these cases the knowledge of the artists' technique, the general artistic practice of the period and which other organic materials are present in the sample can be extremely useful to interpret the results [28,38]. Finally, the table reports the sugar content of saccahride materials that could be source of contamination of the paint sample such as fungi and bacteria, or more importantly wood [39,40].

The analysis of artworks that have been applied to wood, or other cellulose based supports, such as canvas or paper, is especially difficult as the wood degrades overtime, and the sugars can migrate to the painted surface causing contamination. In addition, it seems reasonable to suppose that the dust that normally accumulates on works of art, particularly in objects that are not stored in protected environments, might contain non negligible amounts of wood particles. This may be an explanation for the high content of xylose and glucose found in many samples of works of art, rather than any transformation of the sugars originally present in the sample [28,29,41].

In the light of this new database, the sugar profiles obtained from the analysis of the samples collected from the mural paintings of the Nefertari tomb may be ascribed to an arabic gum obtained from the sap of *Acacia giraffe*, *Acacia karoo*, or *Acacia seyal* trees. The sample from the polychrome wood in Peru shows a sugar profile similar to that of fruit tree gum. Despite this, this hypothesis must be rejected, as fruit trees are not native of the area. As a general rule, the geographical origin of a work of art may help to limit the number of the possible sources of plant materials that were available to the artist. It is thus important to collect reference plant gums from the surrounding area to broaden the database available. Also, knowing when the artwork was created may help to isolate species of plants to the geographical origin. This type of information is especially important for ancient works of art, or when cultures were geographically isolated. Making the hypothesis that the wood of the support of the Peruvian painting might have degraded and its sugars might have migrated into the paint layers, then xylose, glucose and mannose could be ascribed to the wood contamination and the sugar profile observed to a gum from mesquite tree or cashew gum, which are native of the geographical area.

## Conclusions

This paper compares two GC-MS analytical procedures used for the quantitative determination of sugars in polysaccharide materials at the Getty Conservation Institute in Los Angeles, USA (GCI) and the Department of Chemistry and Industrial Chemistry of the University of Pisa, Italy (DCCI). The optimisations of the analytical steps were necessary in order to obtain a reliable sugar profile of polysaccharides in paint samples. The comparison of the independently obtained data of the raw materials, reference paint layers and paint samples showed that the two procedures produce highly comparable results, even when extremely complex systems are taken into consideration.

Given these results, a common database of sugar profiles was built by analysing materials containing a major or minor saccharide fraction, which can be commonly found in a paint sample. The database represents an important step forward in the problem of identifying saccharide materials in art objects, as it highlights the variety of sources of saccharides that can be encountered and the necessity of understanding their contributions when analysing the sugar profile of a paint sample. This was shown by two examples of paintings which were painted with a saccharide binder, whose sugar profile did not match those of the reference arabic, tragacanth and fruit tree gums, or could not be attributed to any of them for geographical reasons. Based on the new knowledge built on the new database available, a potential interpretation of the sugar profiles of the paint samples is suggested.

## Endnotes

^a^In this comparison only xylose, arabinose, fucose, rhamnose, mannose and galactose were taken into consideration. The uronic acids and fructose were in fact not quantitated with both procedures, and glucose is subject to high environmental contamination levels and for this reason is not used in the literature to identify the source of the plant gum in paint samples.

^b^To note that the also the sugar contents of the analysed samples are highly comparable.

## Abbreviations

GC/MS: Gas chromatography/mass spectrometry; ETSH: Ethantiol; NaN_3_: Sodium azide; BSTFA: *N,O*-bis(trimethylsilyl) trifluoroacetamide; TMCS: Trimethylchlorosilane; LOD: Detection limit; LOQ: Quantitation limit; GCI: Getty conservation institute (USA); DCCI: Department of chemistry and industrial chemistry of the University of Pisa (Italy); RSD: Relative standard deviation.

## Competing interest

The authors declare that they do not have competing interests.

## Authors’ contributions

All authors contributed to data analyses and to finalizing the manuscript. All authors have read and approved the final version.

## Supplementary Material

Additional file 1 **Table S1.** Average relative sugar percentage content pigmented and unpigmented reference samples of arabic, cherry and tragacanth gums obtained with the DCCI and GCI procedures. Click here for file

## References

[B1] Andreotti A Bonaduce I Colombini MP Modugno F Ribechini E Colombini MP, Tassi L Characterisation of natural organic materials in paintings by GC/MS analytical procedures New Trends in Analytical, Environmental and Cultural Heritage Chemistry 2008 Kerala: Transworld Research Network 491

[B2] Mills J White R Organic Chemistry of Museum Objects 1999 2 London: Butterworth-Heinemann

[B3] Colombini MP Andreotti A Bonaduce I Modugno F Ribechini E Analytical strategies for characterizing organic paint media using gas chromatography/mass spectrometry Acc Chem Res 2010 43 715 727 10.1021/ar900185f 20180544

[B4] Ruiz-Matute AI Hernandez-Hernandez O Rodriguez-Sanchez S Sanz ML Martinez-Castro I Derivatization of carbohydrates for GC and GC–MS analyses J Chromatogr B 2011 879 1226 1240 10.1016/j.jchromb.2010.11.013 21186143

[B5] Sanz ML Martinez-Castro I Recent developments in sample preparation for chromatographic analysis of carbohydrates J Chromatogr A 2007 1153 74 89 10.1016/j.chroma.2007.01.028 17257608

[B6] Willför S Pranovich A Tamminen T Puls J Laine C Suurnäkki A Saake B Uotila K Simolin H Hemming J Holmbo B Carbohydrate analysis of plant materials with uronic acid-containing polysaccharides-a comparison between different hydrolysis and subsequent chromatographic analytical techniques Ind Crop Prod 2009 29 571 580 10.1016/j.indcrop.2008.11.003

[B7] Mejanelle P Bleton J Tchapla A Goursaud S Gas chromatography–mass spectrometric analysis of monosaccharides after methanolysis and trimethylsilylation. potential for the characterization of substances of vegetal origin: application to the study of museum objects J Chromatogr Libr 2002 66 845 902

[B8] Bleton J Mejanelle P Sansoulet J Goursaud S Tchapla A Characterization of neutral sugars and uronic acids after methanolysis and trimethylsilylation for recognition of plant gums J Chromatogr A 1996 720 27 49 10.1016/0021-9673(95)00308-8

[B9] Pitthard V Griesser M Stanek S Methodology and application of GC-MS to study altered organic binding media from objects of the Kunsthistorisches Museum, Vienna Ann Chim (Rome, Italy) 2006 96 561 573 10.1002/adic.200690058 17172209

[B10] Pitthard V Griesser M Stanek S Bayerova T Study of complex organic binding media systems on artworks applying GC-MS analysis: selected examples from the Kunsthistorisches Museum, Vienna Macromol Symp 2006 238 37 45 10.1002/masy.200650606

[B11] Schneider U Kenndler E Identification of plant and animal glues in museum objects by GC-MS after cataliytic hydrolysis of the proteins by the use of catioin exchanger, with simultaneous separation from carbohydrates Fresenius J Anal Chem 2001 371 81 87 10.1007/s002160100938 11605764

[B12] Kharbade BV Joshi GP Thin-layer chromatographic and hydrolysis methods for the identification of plant gums in art objects Stud Conserv 1995 40 93 102 10.2307/1506508

[B13] Pitthard V Finch P GC-MS analysis of monosaccharide mixtures as their diethyldithioacetal derivatives: application to plant gums used in art works Chromatographia 2001 53 S317 S321 10.1007/BF02490349

[B14] Colombini MP Ceccarini A Carmignani A Ion chromatography characterization of polysaccharides in ancient wall paintings J Chromatogr A 2002 968 79 88 10.1016/S0021-9673(02)00950-0 12236518

[B15] Singh V Sethi R Tewari A Srivastava V Sanghi R Hydrolysis of plant seed gums by microwave irradiation Carbohydr Polym 2003 54 523 525 10.1016/j.carbpol.2003.05.003

[B16] Singh V Tiwari A Kumari P Tiwari S Microwave-promoted hydrolysis of plant seed gums on alumina support Carbohydr Res 2006 341 2270 2274 10.1016/j.carres.2006.05.021 16806127

[B17] Stephen AM Churms SC Vogt DC Exudate Gums Methods Plant Biochem 1990 2 483 522

[B18] MolnaÂ´r-Perl I Role of chromatography in the analysis of sugars, carboxylic acids and amino acids in food J Chromatogr A 2000 891 1 32 10.1016/S0021-9673(00)00598-7 10999622

[B19] Harvey DJ Derivatization of carbohydrates for analysis by chromatography; electrophoresis and mass spectrometry J Chromatogr B 2011 879 1196 1225 10.1016/j.jchromb.2010.11.010 21145794

[B20] Chiantore O Riedo C Scalarone D Gas chromatography–mass spectrometric analysis of products from on-line pyrolysis/silylation of plant gums used as binding media Int J Mass Spectrom 2009 284 35 41 10.1016/j.ijms.2008.07.031

[B21] Riedo C Scalarone D Chiantore O Advances in identification of plant gums in cultural heritage by thermally assisted hydrolysis and methylation Anal Bioanal Chem 2010 396 1559 1569 10.1007/s00216-009-3325-4 20012903

[B22] Andreotti A Bonaduce I Colombini MP Modugno F Ribechini E The diagnosis of the yellowing of the marble high-reliefs and the black decorations in the chapel of the tomb of Saint Anthony (Padua-Italy) Int J Mass Spectrom 2009 284 123 130 10.1016/j.ijms.2008.11.008

[B23] Knapp DR Handbook of Analytical Derivatization Reactions 1979 New York: John Wiley & Sons

[B24] Twilley JW The analysis of Exudate Plant Gums in their artistic applications: An interim report Archaeol Chem 1984 9 357 399

[B25] Birstein VJ On the Technology of Central Asian Wall Paintings: The Problem of Binding Media Stud Conserv 1975 20 8 19 10.2307/1505595

[B26] Wang Z-F He Y Huang L-J An alternative method for the rapid synthesis of partially O-methylated alditol acetate standards for GC-MS analysis of carbohydrates Carbohydr Res 2007 342 2149 2151 10.1016/j.carres.2007.05.028 17585890

[B27] Lluveras A Bonaduce I Andreotti A Colombini MP A GC/MS analytical procedure for the characterization of glycerolipids, natural waxes, terpenoid resins, proteinaceous and polysaccharide materials in the same paint micro sample avoiding interferences from inorganic media Anal Chem 2010 81 376 386 19954203 10.1021/ac902141m

[B28] Ormsby BA Townsend JH Singer BW Dean JR British Watercolour Cakes for the eighteen to the early twentieth Century Stud Conserv 2005 50 45 66

[B29] Bonaduce I Colombini MP Lluveras A Restivo V Ribechini E GC-MS Characterisation of plant gums in samples from painted works of art J Chromatogr A 2007 1175 275 282 10.1016/j.chroma.2007.10.056 18023451

[B30] Brecoulaki H Andreotti A Bonaduce I Colombini MP Lluveras Tenorio A Characterization of organic media in the wall-paintings of the ‘palace of nestor’ at pylos, greece: evidence for a secco painting techniques in the bronze age J Archaeol Sci 2012 39 2866 2876 10.1016/j.jas.2012.04.018

[B31] Rasmussen KL Lluveras Tenorio A Bonaduce I Colombini MP Birolo L Galano E Amoresano A Doudna G Bond AD Palleschi V Constituents of the ink from a Qumran inkwell: New prospects for provenancing the ink on the Dead Sea Scrolls J Archaeol Sci 2012 39 2956 2968 10.1016/j.jas.2012.04.041

[B32] Bonaduce I Cito M Colombini MP Lluveras Tenorio A Petzet M, Jansen M, Emmerling E The characterisation of the organic binders The Giant Buddhas of Bamiyan: safeguarding the remains 2009 Berlin: International Council On Monuments and Sites (ICOMOS) 265 276

[B33] Scott DA Dodd LS Furihata J Tanimoto S Keeney J Schilling MR Cowan E An Ancient Egyptian Cartonnage Broad Collar: Technical Examination of Pigments and Binding Media Stud Conserv 2004 49 177 192 10.2307/25487691

[B34] Scott DA Warmlander S Mazurek J Quirke S Examination of some pigments, grounds and media from Egyptian cartonnage fragments in the Petrie Museum, University College London J Archaeol Sci 2009 36 923 932 10.1016/j.jas.2008.12.011

[B35] Mawhinney TP Feather MS Barbero GJ Martinez JR The rapid, Quantitative Determination of Neutral Sugars (as Aldonitrile Acetates) and Amino Sugars (as O-Mehtyloxime acetates) in glycoproteins by Gas–liquid Chromatography Anal Biochem 1980 110 112 117 7356120 10.1016/0003-2697(80)90048-2

[B36] Idris OHM Williams PA Phillips GO Characterisation of gum from Acacia senegal trees of different age and location using multidetection gel permeation chromatography Food Hydrocolloids 1998 12 379 388 10.1016/S0268-005X(98)00058-7

[B37] del Rey M Wall Paintings of the Tomb of Nefertari: First Quarterly Report 1986 Los Angeles (USA): Getty Conservation Institute

[B38] Lluveras-Tenorio A Mazurek J Restivo A Colombini MP Bonaduce I The developement of a new analytical model for the identification of saccharide binders in paint samples PLoS Onein press 10.1371/journal.pone.0049383 PMC3498129 23166654

[B39] Willför S Sundberg A Hemming J Holmbom B Polysaccharides in some industrially important softwood species Wood Sci Technol 2005 39 245 258 10.1007/s00226-004-0280-2

[B40] Willför S Sundberg A Pravonich A Holmbom B Polysaccharides in some industrially important hardwood species Wood Sci Technol 2005 39 601 617 10.1007/s00226-005-0039-4

[B41] Bleton J Coupry C Sansoulet J Approche d'etude des encres anciennes Stud Conserv 1996 41 95 108 10.2307/1506520

