# Supplementary material for: Analysis of plant gums and saccharide materials in paint samples: comparison of GC-MS analytical procedures and databases
Source: Chem Cent J. 2012 Oct 10;6:115. doi: 10.1186/1752-153X-6-115 (PMC3541984; doi:10.1186/1752-153X-6-115)
Supplement: Additional file 1 — Table S1. Average relative sugar percentage content pigmented and unpigmented reference samples of arabic, cherry and tragacanth gums obtained with the DCCI and GCI procedures. [file 1752-153X-6-115-S1.doc]

Table SM 1. Average relative sugar percentage content pigmented and unpigmented reference samples of arabic, cherry and tragacanth gums obtained with the DCCI and GCI procedures

| **Gum** | **Family and species** | **General origins of plant family** | **Origin** | **notes** | **relative percentage content** | | | | | | | | | | **Procedure3** |
| --- | --- | --- | --- | --- | --- | --- | --- | --- | --- | --- | --- | --- | --- | --- | --- |
|  |  |  |  |  | **xylose** | **arabinose** | **rhamnose** | **fucose** | **galacturonic acid** | **glucuronic acid** | **fructose** | **glucose** | **mannose** | **galactose** |  |
| Arabic | *obtained from the sap of trees Acacia senegal* | Tropical Africa | L. Cornelissen & Son England | homogeneous powdered sample 1 | 0 | 38 | 19 | 0 |  |  | 0 | 0 | 0 | 42 | GCI |
|  |  |  |  | homogeneous powdered sample 2 | 0 | 36 | 18 | 0 |  |  | 0 | 0 | 0 | 46 |  |
|  |  |  |  | paint layer with ultramarine blue 1 | 0 | 44 | 22 | 0 |  |  | 0 | 0 | 0 | 33 |  |
|  |  |  |  | paint layer with ultramarine blue 2 | 0 | 40 | 25 | 0 |  |  | 0 | 0 | 0 | 35 |  |
|  |  |  |  | homogeneous powdered sample 3 | 0 | 35 | 16 | 0 |  |  | 0 | 2 | 0 | 47 |  |
|  |  |  |  | homogeneous powdered sample 4 | 0 | 35 | 16 | 0 |  |  | 0 | 0 | 0 | 48 |  |
|  |  |  |  | homogeneous powdered sample 5 | 0 | 35 | 16 | 0 |  |  | 0 | 0 | 0 | 48 |  |
|  |  |  |  | paint layer with vine black 1 | 0 | 36 | 16 | 0 |  |  | 0 | 1 | 0 | 47 |  |
|  |  |  |  | paint layer with vine black 2 | 0 | 36 | 16 | 0 |  |  | 0 | 0 | 0 | 47 |  |
|  |  |  |  | paint layer with yellow ochre 1 | 0 | 36 | 16 | 0 |  |  | 0 | 1 | 0 | 47 |  |
|  |  |  |  | paint layer with yellow ochre | 0 | 35 | 16 | 0 |  |  | 0 | 1 | 0 | 48 |  |
|  |  |  | Sigma | unpigmented reference paint layer | 0 | 26 | 13 | 0 | 0 | 12 | no | 0 | 0 | 49 | DCCI |
|  |  |  |  | water solution | 0 | 27 | 11 | 0 | 0 | 12 | no | 0 | 0 | 48 |  |
|  |  |  |  | homogeneous powdered sample | 0 | 26 | 13 | 0 | 0 | 12 | no | 0 | 0 | 49 |  |
|  |  |  |  | paint layer with red bole 1 | 0 | 22 | 11 | 0 | 0 | 8 |  | 5 | 0 | 53 |  |
|  |  |  |  | paint layer with red bole 2 | 0 | 28 | 13 | 0 | 0 | 9 |  | 0 | 0 | 50 |  |
|  |  |  |  | paint layer with ultramarine blue 1 | 0 | 31 | 16 | 0 | 0 | 10 |  | 1 | 0 | 42 |  |
|  |  |  |  | paint layer with ultramarine blue 2 | 0 | 27 | 12 | 0 | 0 | 15 |  | 0 | 0 | 46 |  |
|  |  |  |  | paint layer with minium 1 | 0 | 32 | 14 | 0 | 0 | 16 |  | 0 | 0 | 37 |  |
|  |  |  |  | paint layer with minium 2 | 0 | 35 | 17 | 0 | 0 | 8 |  | 0 | 0 | 40 |  |
|  |  |  |  | paint layer on plaster | 0 | 26 | 16 | 0 | 0 | 16 |  | 0 | 0 | 42 |  |
| Cherry  Cherry | *exuded by the trees of the genus Prunus* | Northern Hemisphere | Kremer Pigmente  Germany | homogeneous powdered sample 1 | 11 | 50 | 1 | 0 |  |  | 0 | 1 | 4 | 34 | GCI |
|  |  |  |  | homogeneous powdered sample 2 | 13 | 58 | 1 | 0 |  |  | 0 | 0 | 1 | 26 |  |
|  |  |  |  | paint layer with vine black 1 | 12 | 48 | 2 | 0 |  |  | 0 | 2 | 2 | 35 |  |
|  |  |  |  | paint layer with vine black 2 | 12 | 52 | 1 | 0 |  |  | 1 | 1 | 1 | 32 |  |
|  |  |  |  | paint layer with vermilion 1 | 11 | 48 | 1 | 0 |  |  | 0 | 1 | 1 | 38 |  |
|  |  |  |  | paint layer with vermilion 2 | 10 | 42 | 1 | 0 |  |  | 0 | 2 | 3 | 42 |  |
|  |  |  |  | paint layer with yellow ochre 1 | 10 | 50 | 2 | 0 |  |  | 0 | 0 | 4 | 35 |  |
|  |  |  |  | paint layer with yellow ochre 2 | 11 | 49 | 2 | 0 |  |  | 0 | 2 | 2 | 33 |  |
|  |  |  | Opificio delle Pietre Dure, Florence | unpigmented reference paint layer | 7 | 49 | 2 | 0 | 0 | 6 | no | 0 | 2 | 34 | DCCI |
|  |  |  |  | water solution | 10 | 44 | 2 | 0 | 0 | 6 | no | 0 | 3 | 34 |  |
|  |  |  |  | homogeneous powdered sample | 5 | 35 | 2 | 0 | 0 | 4 | no | 0 | 2 | 52 |  |
|  |  |  |  | paint layer with red bole 1 | 4 | 54 | 1 | 0 | 0 | 12 | no | 0 | 3 | 25 |  |
|  |  |  |  | paint layer with red bole 2 | 3 | 47 | 1 | 0 | 0 | 13 | no | 0 | 3 | 32 |  |
|  |  |  |  | paint layer with ultramarine blue | 7 | 53 | 1 | 0 | 0 | 8 | no | 0 | 3 | 27 |  |
|  |  |  |  | paint layer with minium 1 | 5 | 45 | 1 | 0 | 0 | 3 | no | 0 | 4 | 42 |  |
|  |  |  |  | paint layer with minium 2 | 10 | 41 | 1 | 0 | 0 | 2 | no | 1 | 5 | 41 |  |
| Tragacanth | *obtained from the sap of the plants Astragalus* | Asia | Kremer Pigmente Germany | homogeneous powdered sample 1 | 26 | 43 | 1 | 10 |  |  | 1 | 10 | 0 | 9 | GCI |
|  |  |  |  | homogeneous powdered sample 2 | 26 | 42 | 1 | 10 |  |  | 1 | 11 | 0 | 9 |  |
|  |  |  |  | paint layer with vine black 1 | 26 | 42 | 2 | 10 |  |  | 1 | 10 | 0 | 10 |  |
|  |  |  |  | paint layer with vine black 2 | 25 | 42 | 1 | 10 |  |  | 1 | 10 | 0 | 10 |  |
|  |  |  |  | paint layer with vermilion 1 | 24 | 44 | 2 | 9 |  |  | 1 | 11 | 0 | 10 |  |
|  |  |  |  | paint layer with vermilion 2 | 25 | 44 | 1 | 9 |  |  | 1 | 11 | 0 | 9 |  |
|  |  |  |  | paint layer with ultramarine blue | 19 | 54 | 1 | 16 |  |  | 1 | 4 | 0 | 5 |  |
|  |  |  |  | paint layer with yellow ochre | 25 | 46 | 1 | 11 |  |  | 1 | 8 | 0 | 7 |  |
|  |  |  | Sigma | unpigmented reference paint layer | 21 | 36 | 2 | 7 | 13 | 1 | no | 11 | 0 | 10 | DCCI |
|  |  |  |  | water solution | 19 | 34 | 2 | 8 | 13 | 1 | no | 12 | 0 | 13 |  |
|  |  |  |  | homogeneous powdered sample | 19 | 31 | 2 | 6 | 17 | 1 | no | 12 | 0 | 12 |  |
|  |  |  |  | paint layer with red bole | 26 | 35 | 3 | 11 | 4 | 0 | no | 9 | 0 | 11 |  |
|  |  |  |  | paint layer with ultramarine blue 1 | 22 | 43 | 3 | 9 | 0 | 1 | no | 9 | 0 | 13 |  |
|  |  |  |  | paint layer with ultramarine blue 2 | 21 | 35 | 2 | 7 | 7 | 1 | no | 11 | 0 | 14 |  |
|  |  |  |  | paint layer with minium | 28 | 29 | 2 | **6** | 1 | 0 | no | **22** | **0** | 12 |  |
